# A TCRβ Repertoire Signature Can Predict Experimental Cerebral Malaria

**DOI:** 10.1371/journal.pone.0147871

**Published:** 2016-02-04

**Authors:** Encarnita Mariotti-Ferrandiz, Hang-Phuong Pham, Sophie Dulauroy, Olivier Gorgette, David Klatzmann, Pierre-André Cazenave, Sylviane Pied, Adrien Six

**Affiliations:** 1 Sorbonne Universités, UPMC Univ Paris 06, URA 1961 CNRS, F-75005, Paris, France; 2 CNRS, URA 1961 CNRS, F-75005, Paris, France; 3 Institut Pasteur, Immunophysiopathologie infectieuse, F-75015, Paris, France; 4 Sorbonne Universités, UPMC Univ Paris 06, UMR 7211, UPMC Immunology- Immunopathology-Immunotherapy, F-75013, Paris, France; 5 Inserm, U959, Immunology-Immunopathology-Immunotherapy, F-75013, Paris, France; Oswaldo Cruz Institute (IOC-Fiocruz), BRAZIL

## Abstract

Cerebral Malaria (CM) is associated with a pathogenic T cell response. Mice infected by *P*. *berghei* ANKA clone 1.49 (PbA) developing CM (CM^+^) present an altered PBL TCR repertoire, partly due to recurrently expanded T cell clones, as compared to non-infected and CM^-^ infected mice. To analyse the relationship between repertoire alteration and CM, we performed a kinetic analysis of the TRBV repertoire during the course of the infection until CM-related death in PbA-infected mice. The repertoires of PBL, splenocytes and brain lymphocytes were compared between infected and non-infected mice using a high-throughput CDR3 spectratyping method. We observed a modification of the whole TCR repertoire in the spleen and blood of infected mice, from the fifth and the sixth day post-infection, respectively, while only three TRBV were significantly perturbed in the brain of infected mice. Using multivariate analysis and statistical modelling, we identified a unique TCRβ signature discriminating CM^+^ from CTR mice, enriched during the course of the infection in the spleen and the blood and predicting CM onset. These results highlight a dynamic modification and compartmentalization of the TCR diversity during the course of PbA infection, and provide a novel method to identify disease-associated TCRβ signature as diagnostic and prognostic biomarkers.

## Introduction

Cerebral malaria (CM) represents a global health disease caused by *Plasmodium falciparum* infection. Despite efforts made in controlling *Plasmodium* infection spreading in the last decade, its burdens remains extensive, endemically accounting for 30% of the 627,000 infection-related deaths as estimated in 2012 [1]. CM is obviously associated with neurological features caused by the binding onto endothelial cells (EC) *of P*. *falciparum* parasitized red blood cells (pRBC) leading to their sequestration in the brain microvessels [2,3]. Intravascular leukocytes and platelets infiltration has been also observed in brains of Malawian children dead of CM [4]. Mouse models of experimental CM (ECM), greatly contribute to the description of the immune response in cerebral malaria, confirmed the major role of T lymphocytes in the neuropathogenesis [5]. Particularly, this emphasizes the major implication of both CD4^+^ and CD8^+^ Tαβ cells in the development of ECM [6–11]. Indeed, we and others observed the sequestration of Tαβ lymphocytes in the brain of mice developing CM (CM^+^) [12,13]. However, evidence regarding the natural antigenic specificities of these infiltrating T cells is still poor. Studies using recombinant parasite constitutively expressing the OVA peptide showed that pathogenic CD8^+^ T cells reaching the brain are specific for this exogenous peptide [14], confirming that “parasite-specific” CD8^+^ T cells are induced during infection. Very recently, two groups identified PbA epitopes recognized by different CD8^+^ T cells, each of which being characterized by different T cell receptors (TCR), which in turn reach and might damage the brain [15–17]. Although, most of those epitopes are associated with an enrichment of specific CD8^+^ T cells in both spleen and brain of PbA infected mice, none of them can protect mice from ECM outcome. Given that *Plasmodium* is characterized by a differential pattern of protein expression through his life-cycle and a high diversity of molecules, including antigen, superantigen and mitogen [18–21], it is conceivable that ECM outcome is the results of a synergic action of these several molecules leading to inappropriate responses that, in turn, scramble or divert the protective appropriate response. As a consequence, T cell repertoire might be profoundly altered in contrast with a more classical restricted clonal response.

In fact, we previously showed that blood TCRβ repertoire of CM^+^ mice is greatly perturbed compared to healthy mice and also to infected mice without cerebral symptoms. This perturbation is partly due to recurrently expanded T cell clones [22]. However, it remains unclear whether those modifications are the cause or the consequence of the disease. In order to address the quality of lymphocyte responses during the course of experimental CM infection, we described their antigen-specific receptor diversity, produced by somatic DNA rearrangements of V, (D) and J segments later spliced to C segments [23], using CDR3 spectratyping and the ISEApeaks strategies [24–26] on blood and spleen lymphocytes, from day 3 post-infection (p-i) until the ECM-related death of PbA-infected mice. Additionally, we characterized the whole brain Tαβ cell repertoire in naïve and PbA-infected mice. Using a microarray-derived analysis and prediction modelling, we looked for TCRβ peak signatures. Our results showed that splenic and blood TCRβ repertoires are progressively and broadly modified simultaneously with disease development with spleen modifications appearing before blood modifications. Importantly, we explored the whole TCRαβ repertoire in mouse brain and we showed that, although peculiar in naïve mice, there are few but major modifications following the infection, suggesting a particular response in the brain of PbA-infected mice. Finally, we identified a list of TCRβ peaks forming a signature associated with ECM development and appearing during the course of the infection. Altogether, these data strongly support the idea that T cell diversity as a whole must be taken into account for drug and vaccine development against infectious diseases.

## Material and Methods

### Mice and Parasites

Seven-week-old B10.D2 mice were purchased from Harlan UK Limited one week before infection and housed in filter-topped cages under specific pathogen-free conditions in the Institut Pasteur animal facilities of the Immunulogy Department at Institut Pasteur (Paris, France), under a 12h:12h light:dark cyle, and received the same food and water provided by the animal facility staff. Food and water bottle were changed daily and made available 24h a day for all the animals of the same cage during the whole experiment timeline. Up to five sex-matched mice were grouped per cage and followed during the experiment timeline. The clone 1.49L of *Plasmodium berghei* ANKA was kindly given by Dr. Walliker (Institute of Genetics, Edinburg, UK) and is maintained in our laboratory on C57BL/6J female mice. This clone induces in mice a neurological syndrome partly mimicking the one of human CM. Blood stages of the parasite were cryopreserved in liquid nitrogen as stabilates in Alserver’s solution containing 10% glycerol.

### Infection

Mice infection was induced in 75 mice by intraperitoneal injection of 10^6^ parasitized Red Blood Cells (pRBC). 35 mice were sacrificed at day 3 (n = 5), 4 (n = 10), 5 (n = 10) and 6 (n = 10) post-infection, constituting four groups for the kinetic study. A total of 40 mice were sacrificed at the stage of cerebral malaria later referred to as CM^+^. Animals under continuous human observation were classified as CM+ when decreased body temperature and ataxia or hemiplegia or paraplegia or convulsions were recorded. Animals may have experienced the suffering associated with CM+ signs, though not excessive since they were sacrificed whenever diagnosed as such. To prevent excessive suffering, we implemented a shift of 2 people every 8 hours (3 groups of 2 people per 24h) to ensure human presence, animal observation and handling during the whole experiment duration. To minimize animal suffering, animals where under continuous observation 24h per day during the whole experiment duration by 2-people shifts every 8h, immediately sacrificed whenever diagnosed with CM+ signs and handled according to recommended regulations. At the same time, 24 B10.D2 mice which received either PBS or uninfected C57BL/6 Red Blood Cells (RBC) were sacrificed to constitute the control group (CTR). Parasitemia was measured on Giemsa-stained thin blood smears from day 4 p-i for all infected mice. All individuals included in this study displayed parasite positive staining from day 4 until ECM development, with 1 to 20% of parasitized RBCs (data not shown).

### Ethics Statements

Institut Pasteur animal facility was accredited by the French Ministry of Agriculture to perform experiments on live mice, in application of the French (Decree 87–848 issued on 19/10/1987) and European (Directive 86/609/CEE) regulations on care and protection of Laboratory Animals. All animal experiments were approved and conducted in accordance with the Institut Pasteur Biosafety Committee (Paris) and performed in compliance with the NIH Animal Welfare Assurance #A5476-01 issued on 02/07/2007. All efforts were made to minimize animal suffering, mice euthanasia was performed using Carbon Dioxide flow in chamber for 3 min.

### Cell Preparation

For each group (CTR, CM^+^) and subgroups (day3, 4, 5 and 6 p-i), blood and spleen were removed. Blood was obtained on heparin by retroorbital or intracardiac punction. Mononuclear cells were isolated on Ficoll-Hypaque gradient (Pharmacia, France). Spleen was removed and cells suspended in 3% FCS-PBS. Brain was harvested from CTR, day 6 p-i and CM^+^ animals and maintained for thirty minutes in Hepes medium containing 0.05% of Collagenase (Sigma). Cells were then isolated on 30% Percoll gradient in DMEM medium and suspended in 3% FCS-PBS. Residual RBCs were removed from all the samples by hypotonic shock using ammonium chloride (ACK) lysis buffer for 1 to 2 minutes at room temperature. Cell preparations were then washed twice with 3% FCS-PBS. Lymphoid cells were counted using Malassez cell in presence of eosin to exclude dead cells.

### TCRB Repertoire

In order to avoid bias, samples were treated randomly. Depending on the sample, total RNA was extracted from 250,000 to 8.10^6^ mononuclear cells using the TRI REAGENT kit (Molecular Research Center, Cincinnati, Ohio). 20 μg of glycogen (Roche, Meylan, France) was used to ensure optimal precipitation of RNA and pellet visualization. cDNA was synthesised for 1h30 at 42°C using retrotranscriptase for AM virus (Roche-Boehringer) and an inhibitor of RNAse (RNAsin, Promega) was added to avoid RNA degradation. PCR were done using cDNA corresponding to 250,000 mononuclear cells. Protocols for TCR TRBV-TRBC CDR3 spectratyping have already been explained elsewhere in detail [22,24]. TRBC and TRBV primer sequences were as described earlier [22]. As TRBV12-3 [27], TRBV24 [28] and TRBV21 [29] are not functional in B10.D2 mice, they were not analysed. TRBV16 was excluded from this study afterwards due to technical problem with the corresponding primer. PCR products were loaded on a 36-well ABI373 or 96-well ABI377 automated sequencer (Applied Biosystems, Foster city, CA) and separated according to their nucleotide length forming a profile of peaks for each primer combination, spaced by 3 nucleotides as expected for in-frame transcripts. Each peak corresponds to a CDR3 length. The Immunoscope software [24] was used to obtain peak area and nucleotide length and CDR3 profile displays from sequencer raw data. IMGT nomenclature has been used for TRBV genes [30].

### CDR3 Spectratype Analysis

We used the ISEApeaks software package (2000–2002 Institut Pasteur, Paris, France) to extract, smooth, manage and analyse the data [25,26]. For each CDR3 length profile, the peak distribution is calculated as the percentage of each peak, obtained by dividing its area by the total area of all peaks within the profile. Briefly, for each TRBV-TRBC combination analysed, a reference repertoire is computed as the average CDR3 peak distribution of the samples belonging from the control group. Then, the distance between the peak distribution of each sample and that of the reference repertoire is calculated for each TRBV-TRBC combination. This distance, named DBV-BC, is the perturbation index reflecting the perturbation of the repertoire against a reference repertoire [31,32]. Since perturbation score distributions are often skewed, we log-transformed them for statistical analysis. Then, for each sample of each experimental group, the average of the DBV-BC (μDBV-BC) perturbations of all the TRBV-TRBC combinations is calculated, reflecting the global perturbation of the repertoire. DBV-BC perturbations indices range from 0 (identical profiles) to 100 (completely different profiles). To deal with possible missing values while keeping statistical power for unbiased conclusions, we applied multiple imputation as proposed by Rubin et al. [33]: (1). Missing data were imputed randomly 1000 times to produce 1000 complete datasets using predictive mean matching (ppm) method [34,35]; (2). On each of 1000 complete datasets, linear regressions were used to model the relationship between perturbation scores and day post-infection for each TRBV; (3). Estimated regression coefficient (or slope) and its standard error were pooled from 1000 analyses to give a final result; (4). A test is performed to assess whether the regression coefficient is significantly different from 0.

Multivariate methods were used to analyse data such as Principal Component Analysis (PCA) and unsupervised clustering methods of perturbation scores to study globally the modification of repertoire between organs and after infection and explore the underlying structure of data. We defined global perturbation index as the average perturbation score across all TRBV (μμDBV-BC). ANOVA test was used to compare the global perturbation index within days post-infection and organ. To control the false discovery rate in multiple testing, we adjusted p-values using Benjamini-Hochberg’s method [36]. Statistical significant level was fixed at α = 0.05 (type I error: the probability to reject the null hypothesis when it is true).

### TCRβ Peak Signature Discovery

Gene set enrichment analysis, a microarray-based method, allows to determine whether an a priori gene set is significantly enriched in one of two biological conditions. For each organ, TCRβ peaks were first ranked according to t-statistic based on pooled coefficient computed for differential expression between uninfected and CM^+^ mice by multiple imputation process as described above. For each signature, GSEA provided normalized enrichment score, p-value and q-value for multiple testing problem (details on how GSEA works are reported in Subramanian et al. [37]). TCRβ peak sets or clusters were formed using pvclust, an unsupervised bootstrap-based version of hierarchical classification tree [38], which assesses p-values indicating how strong are the cluster. Significantly enriched clusters identified by GSEA are called signatures. To test the predictive power of significantly enriched signatures, random forest models were trained on our datasets and cross-validated on an independent dataset (24). Missing datasets were imputed 100 times to produce 100 complete datasets using the above imputation process. A random forest model was trained on each of the 100 complete datasets with 100 trees each (intermediate models) and combined within a single model (final model). This model was tested on a test dataset. For cross-validation, random forest model was trained on the test dataset and tested on the training dataset. Prediction accuracy was set as the good prediction rate based on the confusion matrix (**S1 Fig**).

### Statistical Analysis Tools

Statistical analysis was performed using R platform v3.0.2 with the following packages in addition: mice 2.17 [39], ade4 1.5–2, survival 2.37–4, pvclust 1.2–2, randomForest 4.6–7 [40]. These tools are available at CRAN repository http://www.r-project.org. GSEA 2.0.13 software was installed from http://www.broadinstitute.org/gsea.

## Results

### Compartmentalized Repertoire Diversity during PbA Infection

**Fig 1:**
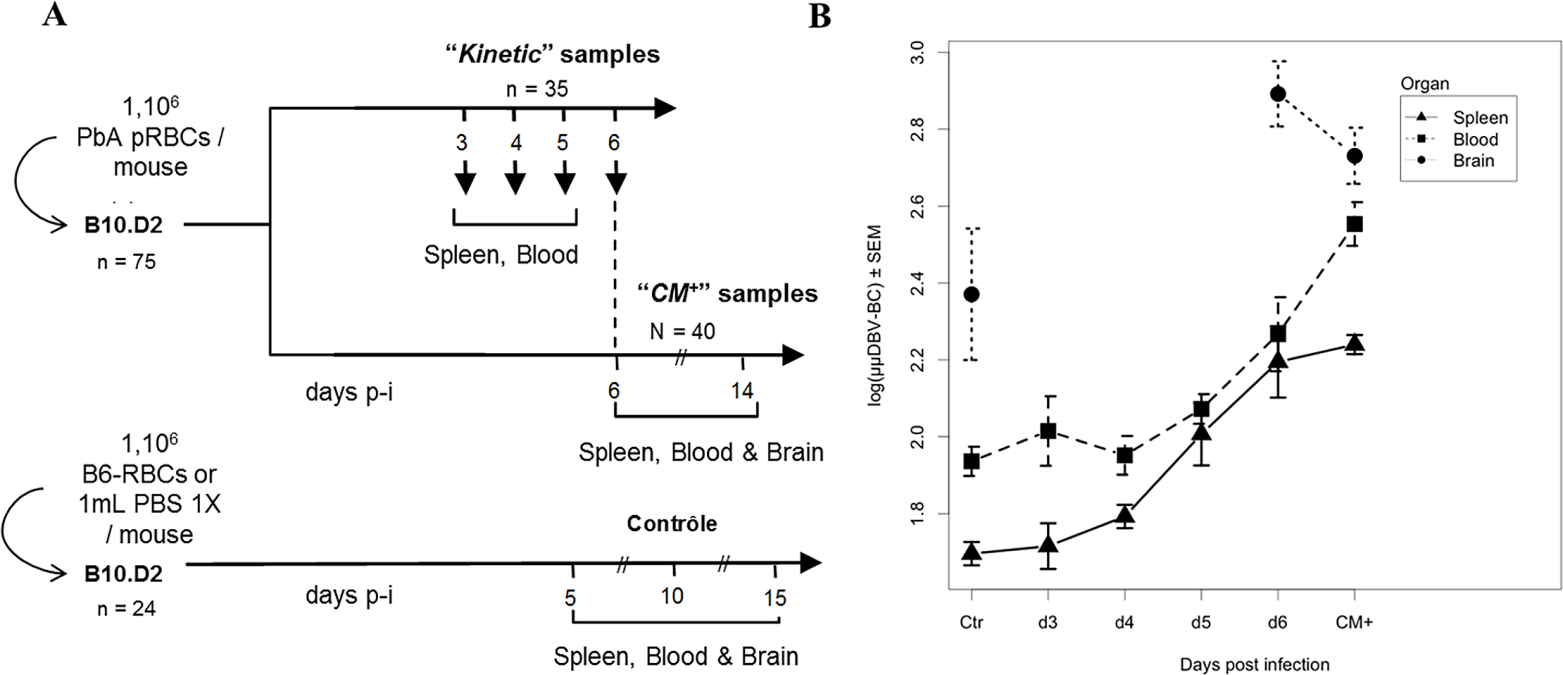
Kinetic analysis of the TCR TRBV-TRBC repertoire during the course of PbA infection. (A) Experimental procedure showing the preparation of samples from both infected and non-infected mice, as well as the parallel analysis of mice during the course of the infection (“kinetic” samples) and at the time of ECM onset (“CM+” samples). Days post-infection (days p-i) indicate the time at which animals of the corresponding groups are sacrificed. Organs harvested for each group are indicated. (B) Modification of the TRBV-TRBC repertoire in spleen, blood and brain of B10.D2 mice during the course of *Plasmodium berghei* ANKA infection. Average DBV-BC perturbations across all TRBVs and individuals in each group (μμDBV-BC) in the spleen (black), the blood (red) and the brain (green) are shown for the control uninfected (CTR), day 3 p-i (d3), day 4 p-i (d4), day 5 p-i (d5), day 6 p-i (d6) and CM+ groups. DBV-BC perturbations were computed with ISEApeaks using CTR Spleen as the reference group.

**Fig 2:**
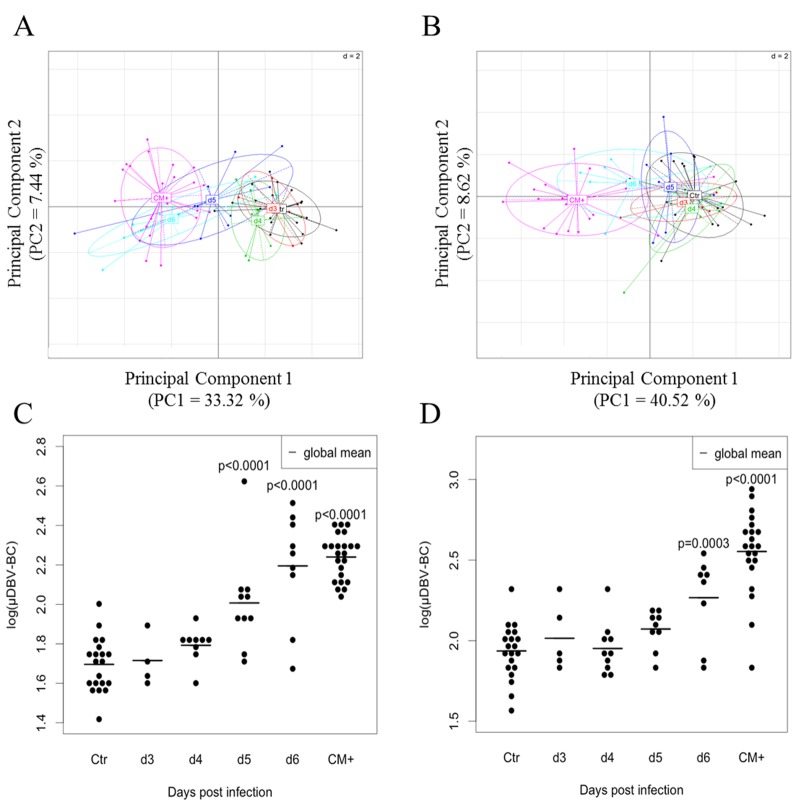
Differential kinetics of TCR TRBV-TRBC repertoire perturbation in the spleen and blood of infected mice. (A-B) Kinetic representation of global perturbation scores across all TRBVs in the spleen (A) and the blood (B) using principal component analysis (PCA). Progressive modification of the repertoire is diagrammed by the shift of day p-i-related groups from the right to the left on the first PCA component (PC1). Colors correspond to analyzed groups. (C-D) Mean DBV-BC perturbations across all TRBVs (μDBV-BC) in the spleen (C) and the blood (D). DBV-BC were computed as in Fig 1B. Black dots represent individual mouse global perturbation scores. Red dots represent the average global perturbation score for each group. Statistical comparisons were performed using the two-way ANOVA test for the difference between organs and between infected groups. Tests were significant for organs (p<0.0001) and day post infection (p<0.0001) at α = 0.05.

Our previous study showed that blood Tαβ cell repertoire is highly perturbed in PbA-infected B10.D2 mice developing ECM (CM^+^) [22]. We aimed at determining whether these modifications occur in the early days before the disease and could be used as a signature of the development of ECM in mice. For this purpose, we analysed the TCRβ diversity of B10.D2 mice from day 3 until ECM-related death. As described in Material and Methods and in **Fig 1A**, we infected 75 mice with 1.10^6^ PbA pRBC, among which 35 were sacrificed at day 3 (5 mice), 4 (10 mice), 5 (10 mice) and 6 (10 mice) post-injection (p-i) constituting four groups for the kinetic study and 40 mice were killed when they developed ECM (as described in material and methods), constituting the CM^+^ group. The control uninfected group was composed by 24 mice half of which received PBS or 1.10^6^ non-parasitized RBCs. Spleen, blood and brain were harvested as described in Material and Methods, leucocytes were isolated and following RNA extraction and cDNA synthesis, we performed the combined CDR3 spectratyping technique and ISEApeaks strategy in order to evaluate the perturbation of the repertoire. Perturbation score was calculated against the average repertoire of the spleen control uninfected group as the polyclonal reference repertoire for all the groups (see Material and Methods and [26,31]). Perturbation score distributions were log-transformed and multiple imputation was applied to correct for bias due to missing data. Descriptive analyses were performed on the average of 1000 complete datasets. **Fig 1B** represents the average of global perturbation score (μμDBV-BC) of splenocytes, PBLs and brain repertoires during the course of infection. The perturbation increased in all organs compared to the organ related CTR groups and was different between organs (two-way ANOVA p-values showed significant perturbation between organs (p<0.0001) and between day p-i (p<0.0001)). These results suggest a compartmentalization of the response to PbA infection. We compared, for each compartment, the global perturbation scores of infected groups against the CTR group. In order to explore globally the repertoire modification across all TRBV, we performed a principal component analysis (PCA) on perturbation scores computed on spleen and blood repertoire (**Fig 2**). PCA plots show a separation of day 5, day 6 p-i and CM^+^ groups (negative PCA scores) from CTR, day 3 and day 4 p-i groups (positive PCA scores) in the spleen (**Fig 2A**). In the blood, day 6 p-i was separated from the other groups (**Fig 2B**). These observations were confirmed by t-tests showing that the perturbation index is significantly different from day 5 (p<0.0001) in the spleen (**Fig 2C**) and day 6 (p = 0.0003) in the blood (**Fig 2D**), until the development of ECM (p<0.0001 for both compartments) when compared to CTR group. Finally, the perturbation index is higher in CM^+^ blood compared to CM^+^ spleen (**Fig 2C and 2D**), as shown previously [22]. These results indicate that during infection, the T cell repertoire is modified early in the spleen compared to the blood, suggesting a dynamic with time of the modifications between both compartments.

### Progressive but Massive Modifications of Spleen and Blood Repertoires during PbA Infection

We further analysed the perturbation for each TRBV-TRBC combinations accounting for a total of 2079 variables. As previously, we used a multiple imputation process (see Material and Methods). 1000 complete datasets were generated and linear regressions were fitted for each TRBV-TRBC combination to compare perturbation score in each day p-i to uninfected group. Regression coefficients estimated over complete datasets were pooled to produce a unique coefficient and its confidence interval. Using such information, t-tests were performed to compare each infected group to the CTR in the corresponding organ. Results are shown in the **Table 1**. Except for TRBV14, all other TRBVs are deeply altered. In the spleen, significant alterations were observed at day 5 p-i for 8 out of 20 TRBVs to which 7 other TRBVs were added at day 6 p-i. TRBV31 is transiently perturbed at day 5. In blood, significant perturbations appeared at day 6 for five TRBVs followed by an alteration of the overall repertoire, except for TRBV13-2, TRBV14. Interestingly, before the development of ECM, some TRBVs perturbed in the blood are not perturbed in the spleen, suggesting that the kinetic of the modifications is proper to each compartment, the blood containing circulating cells from the whole body. Altogether, these results show that TCR diversity is highly modified not only in CM^+^ mice but also during the course of PbA infection, supporting the hypothesis that the alteration of the TCRβ repertoire is an upstream and rather early process with regards to disease development, and thus probably a cause of ECM onset. In addition, the alteration of several TRBVs indicates multiple immunogenic sources.

**Table 1:** Estimated regression coefficient and 95% confidence interval of log perturbation values between infected and control groups in spleen and blood.

TRBV	Group	D3	D4	D5	D6	CM+
**1**	**Spleen**	0.07	0.25	0.52	0.66	1.24
		[-0.46;0.6]	[-0.14;0.64]	[0.15;0.9]*	[0.27;1.05]**	[0.94;1.54]***
	**Blood**	-0.06	-0.21	-0.02	0.32	1.16
		[-0.57;0.45]	[-0.62;0.21]	[-0.43;0.39]	[-0.11;0.75]	[0.84;1.47]***
**2**	**Spleen**	0.2	0.3	0.23	0.37	0.61
		[-0.22;0.61]	[-0.01;0.6]	[-0.06;0.53]	[0.07;0.68]*	[0.37;0.84]***
	**Blood**	0.38	0.15	0.36	0.47	0.77
		[-0.04;0.8]	[-0.18;0.47]	[0.03;0.7]	[0.11;0.83]*	[0.5;1.04]***
**3**	**Spleen**	0.09	0.1	0.54	0.72	1.02
		[-0.37;0.56]	[-0.25;0.44]	[0.21;0.87]*	[0.38;1.07]***	[0.76;1.29]***
	**Blood**	-0.05	0.18	0.29	0.54	1.05
		[-0.48;0.38]	[-0.16;0.53]	[-0.05;0.64]	[0.18;0.9]*	[0.78;1.32]***
**4**	**Spleen**	0.15	0.4	0.2	0.75	0.52
		[-0.38;0.68]	[0.01;0.79]	[-0.19;0.59]	[0.36;1.14]**	[0.22;0.82]**
	**Blood**	0.15	0.3	0.1	0.3	0.73
		[-0.31;0.6]	[-0.06;0.66]	[-0.26;0.46]	[-0.08;0.68]	[0.45;1.01]***
**5**	**Spleen**	0.16	0	0.37	0.56	0.68
		[-0.27;0.59]	[-0.32;0.31]	[0.07;0.68]*	[0.25;0.88]**	[0.44;0.93]***
	**Blood**	-0.3	-0.13	-0.41	0.01	0.59
		[-0.67;0.07]	[-0.43;0.17]	[-0.71;-0.12]	[-0.31;0.33]	[0.35;0.82]***
**12–1**	**Spleen**	-0.41	-0.15	0.03	0.31	0.14
		[-0.9;0.09]	[-0.51;0.21]	[-0.32;0.38]	[-0.05;0.67]	[-0.14;0.41]
	**Blood**	0.11	0.06	0.56	0.32	0.5
		[-0.29;0.52]	[-0.29;0.4]	[0.21;0.9]*	[-0.07;0.7]	[0.23;0.77]***
**12–2**	**Spleen**	-0.18	-0.31	0.43	0.45	0.56
		[-0.72;0.37]	[-0.78;0.17]	[0.01;0.85]	[0.03;0.88]	[0.21;0.91]**
	**Blood**	-0.03	0.1	0.49	0.46	0.76
		[-0.6;0.54]	[-0.37;0.57]	[0.03;0.96]	[0.03;0.89]	[0.38;1.14]***
**13–1**	**Spleen**	-0.22	-0.37	-0.16	0.22	0.28
		[-0.65;0.22]	[-0.69;-0.05]	[-0.47;0.15]	[-0.1;0.54]	[0.03;0.52]*
	**Blood**	-0.25	-0.01	0.21	0.03	0.61
		[-0.71;0.21]	[-0.37;0.35]	[-0.18;0.59]	[-0.36;0.42]	[0.32;0.9]***
**13–2**	**Spleen**	0.12	0.38	0.27	0.59	0.41
		[-0.38;0.62]	[0.02;0.75]	[-0.09;0.62]	[0.22;0.96]**	[0.12;0.69]**
	**Blood**	0.22	0.07	0.26	0.31	0.19
		[-0.19;0.63]	[-0.25;0.39]	[-0.07;0.6]	[-0.05;0.67]	[-0.07;0.45]
**13–3**	**Spleen**	0	0.05	0.15	0.4	0.49
		[-0.4;0.4]	[-0.24;0.35]	[-0.14;0.43]	[0.1;0.69]*	[0.27;0.72]***
	**Blood**	0.24	0.02	0.09	0.37	0.43
		[-0.18;0.65]	[-0.3;0.34]	[-0.25;0.42]	[0.01;0.73]	[0.17;0.69]**
**14**	**Spleen**	-0.13	0	0.17	0.07	0.05
		[-0.45;0.19]	[-0.24;0.23]	[-0.07;0.42]	[-0.17;0.3]	[-0.14;0.23]
	**Blood**	-0.04	-0.08	-0.1	0.03	-0.06
		[-0.35;0.26]	[-0.32;0.17]	[-0.35;0.15]	[-0.23;0.3]	[-0.26;0.14]
**15**	**Spleen**	-0.17	0.16	0.17	0.26	0.73
		[-0.69;0.35]	[-0.24;0.56]	[-0.19;0.54]	[-0.13;0.66]	[0.43;1.02]***
	**Blood**	-0.18	-0.26	0.17	0.58	0.61
		[-0.67;0.31]	[-0.64;0.12]	[-0.23;0.56]	[0.15;1.01]*	[0.29;0.92]***
**16**	**Spleen**	0.09	-0.02	0.12	0.05	0.11
		[-0.24;0.42]	[-0.27;0.23]	[-0.12;0.36]	[-0.19;0.3]	[-0.07;0.3]
	**Blood**	-0.27	-0.17	-0.29	-0.07	0.11
		[-0.58;0.03]	[-0.41;0.08]	[-0.55;-0.04]	[-0.32;0.19]	[-0.08;0.3]
**17**	**Spleen**	0.4	0.34	0.24	0.19	0.5
		[-0.13;0.93]	[-0.09;0.76]	[-0.14;0.61]	[-0.19;0.57]	[0.19;0.81]**
	**Blood**	0.2	-0.06	0.07	0.07	0.36
		[-0.29;0.68]	[-0.45;0.33]	[-0.35;0.49]	[-0.34;0.48]	[0.04;0.68]*
**19**	**Spleen**	0.2	0.19	0.49	0.64	0.55
		[-0.27;0.67]	[-0.15;0.54]	[0.14;0.83]*	[0.3;0.98]**	[0.29;0.81]***
	**Blood**	-0.02	0.37	0.17	0.7	0.56
		[-0.45;0.41]	[0.04;0.71]	[-0.18;0.52]	[0.33;1.06]**	[0.29;0.83]***
**20**	**Spleen**	-0.2	0.17	0.44	0.63	0.44
		[-0.69;0.29]	[-0.19;0.53]	[0.09;0.79]*	[0.26;0.99]**	[0.16;0.73]**
	**Blood**	0.38	0.14	-0.12	0.1	0.63
		[-0.16;0.91]	[-0.28;0.56]	[-0.58;0.33]	[-0.37;0.56]	[0.28;0.98]**
**23**	**Spleen**	0.24	0.38	0.43	0.55	0.61
		[-0.21;0.68]	[0.05;0.71]	[0.12;0.74]*	[0.22;0.87]**	[0.36;0.85]***
	**Blood**	-0.02	0.06	0	0.35	0.63
		[-0.5;0.45]	[-0.31;0.43]	[-0.38;0.38]	[-0.04;0.75]	[0.32;0.95]***
**26**	**Spleen**	0.15	0.03	0.75	1.05	0.75
		[-0.29;0.59]	[-0.3;0.36]	[0.44;1.07]***	[0.72;1.38]***	[0.5;1]***
	**Blood**	0.3	-0.25	0.03	0.7	1.24
		[-0.19;0.8]	[-0.63;0.13]	[-0.39;0.44]	[0.29;1.11]*	[0.92;1.55]***
**29**	**Spleen**	-0.34	-0.22	0.09	0.5	0.56
		[-0.85;0.18]	[-0.6;0.15]	[-0.28;0.45]	[0.12;0.87]*	[0.27;0.85]***
	**Blood**	0.04	-0.11	0.17	0.49	0.74
		[-0.41;0.48]	[-0.43;0.2]	[-0.15;0.5]	[0.12;0.85]*	[0.48;0.99]***
**30**	**Spleen**	0.33	0.16	0.37	0.71	0.28
		[-0.18;0.83]	[-0.22;0.53]	[0.01;0.73]	[0.34;1.09]**	[-0.01;0.58]
	**Blood**	0.21	-0.06	0.25	0.42	0.36
		[-0.3;0.71]	[-0.46;0.35]	[-0.14;0.64]	[0;0.85]	[0;0.71]
**31**	**Spleen**	-0.09	0.08	0.51	0.34	0.41
		[-0.55;0.37]	[-0.26;0.41]	[0.18;0.84]*	[-0.01;0.68]	[0.16;0.67]**
	**Blood**	0.3	0.02	0.11	0.03	0.49
		[-0.19;0.79]	[-0.37;0.4]	[-0.3;0.51]	[-0.38;0.44]	[0.18;0.8]**

*** adjusted p<0.001

** adjusted p<0.01

* adjusted p<0.05

### Targeted T Cell Repertoire Modifications in the Brain of PbA-Infected Mice

**Fig 3:**
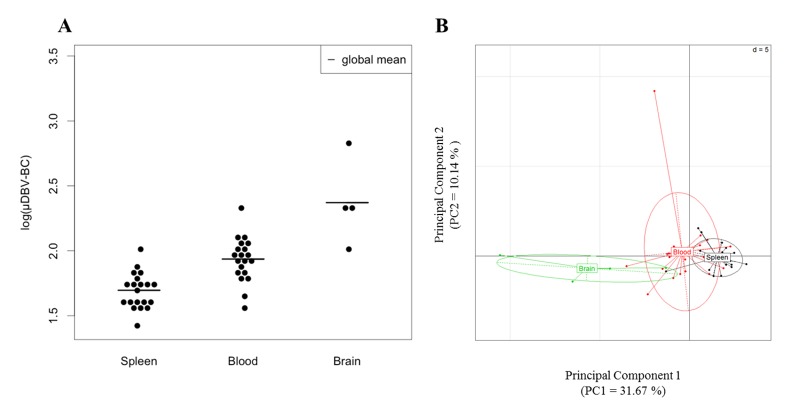
TRBV-TRBC repertoire differences in spleen, blood and brain of naïve B10.D2 mice. (A) Mean DBV-BC perturbations across all TRBVs (μDBV-BC) in the spleen (left), blood (center) and brain (right) of CTR uninfected mice are shown for each individual. DBV-BC perturbations were computed with ISEApeaks using CTR Spleen as the reference group. Statistical comparisons were performed using pairwise t-test with correction for FDR between groups depicting significant p-value (p<0.0001) for each comparison. (B) PCA on DBV-BC log perturbation scores separating repertoires of the spleen, blood and brain on the x axis (PC1).

Next, we wanted to characterize the diversity of the brain T cell population repertoire. The perturbation index was calculated as previously using CTR Spleen as the reference repertoire. First, we focused our attention on the CTR group. Only a limited number of T cells (2 to 3.10^4^ cells) are found in the brain of naïve mice [12]. In order to characterize the whole repertoire, we pooled six brains of naïve mice per control sample to ensure enough material for full TCRβ repertoire analysis and retain the representative range of daily circulating brain T cell population [41]. We performed pairwise comparisons by t-test to confront perturbation scores of CTR brain against CTR spleen and CTR blood and found that perturbation scores of CTR groups are significantly different from each other (**Figs 3A and 2B**, adjusted p<0.0001 for each comparison). This result revealed a clear compartmentalization of the T cell repertoire already at steady-state and a higher inter-individual variability of blood TRBV.

**Fig 4:**
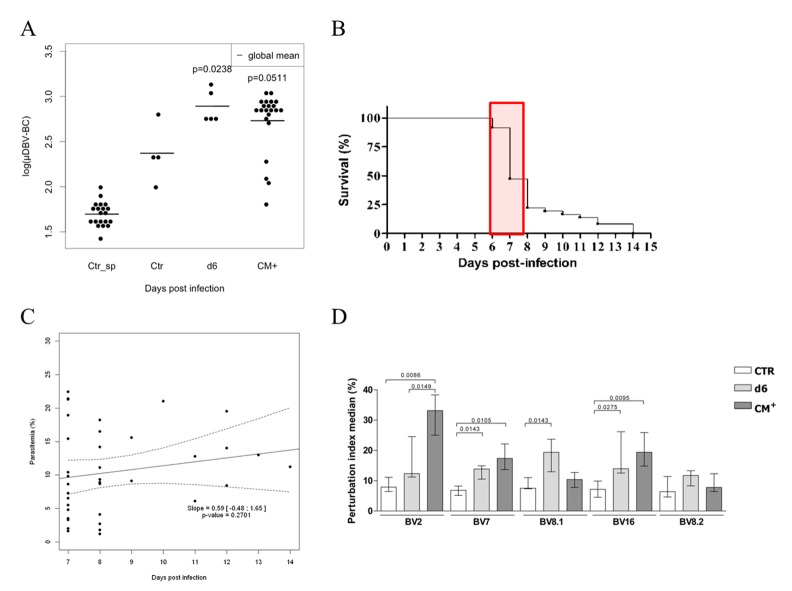
Modification of the TRBV-TRBC repertoire in the brain of B10.D2 mice during the course of PbA infection. (A) Mean DBV-BC perturbations across all TRBVs (μDBV-BC) in the brain of CTR uninfected (CTR), day 6 p-i (Day 6) and CM^+^ mice are shown for each individual. DBV-BC were computed as in Fig 1B. Black dots represent individual mouse scores. Lines represent the average score for each group. Average DBV-BC of CTR spleen is indicated as the reference (Ctr_sp). (B) Survival curve of B10.D2 mice infected with 10^6^ PbA-PRBC. All mice developed ECM symptoms. 75% of the mice died between day 6 and day 8. (C) Regression slope and 95% confidence band of parasitemia (%) over day post infection. The infection has no effect on the % of parasitemia (no significant slope). (D) DBV-BC perturbations scores of five TRBV. Statistical tests were performed as described in Fig 2.

We therefore analysed the global perturbation score of the brain T cell repertoire of CTR and infected mice (at day 6 p-i as well as CM^+^ mice). Globally, day 6 and CM^+^ brain repertoires are significantly different compared to CTR Spleen (p<0.0001) moreover, a significant perturbation between day 6 p-i, but not CM^+^, and uninfected brain repertoire was also detected (p = 0.0238) (**Fig 4A**). The high inter-individual variability of CM^+^ brain sample repertoires might explain these inconsistent results. To ensure that this variability was not due to the large timeframe of ECM onset as shown in **Fig 4B**, we subdivided the CM^+^ group into two groups: CM^+^ mice identified between day 6 and 8 p-i and CM^+^ identified between day 10 and 14, given that all the CM^+^ mice died after similar clinical manifestations. Global perturbation scores calculated for both groups showed no significant differences (data not shown). These results are consistent with the comparable level of parasitemia observed for mice developing ECM between day 7 and 14 p-i (**Fig 4C**, not significant regression coefficient; 0 is included in the confidence interval and p = 0.2701). This again strongly supports the hypothesis that important TCR repertoire perturbations are a cause of ECM development. However, another non-exclusive explanation would be that few TRBVs are perturbed in the brain during infection. Since all TRBVs in the brain are significantly perturbed at day 6 p-i and CM^+^ compared to the uninfected spleen group (data not shown), we therefore analysed the perturbation per TRBV in the brain against the uninfected brain group (**Fig 4D**). t-tests on pooled coefficients against uninfected mice showed that three TRBVs are significantly perturbed in infected CM^+^ mice compared to CTR brain: TRBV1 (adjusted p = 0.0044), TRBV29 (adjusted p = 0.0237) and TRBV3 (adjusted p = 0.0237). TRBV13-3 was significantly perturbed at day 6 p-i (adjusted p = 0.007) but not in CM^+^ brain repertoires.

Altogether, our data show that, although there is a massive perturbation of the whole spleen and blood repertoire of infected mice, only three TRBV are perturbed in the brain of infected mice compared to uninfected mice.

### A TCRβ Repertoire Signature Predicts Cerebral Malaria

**Fig 5:**
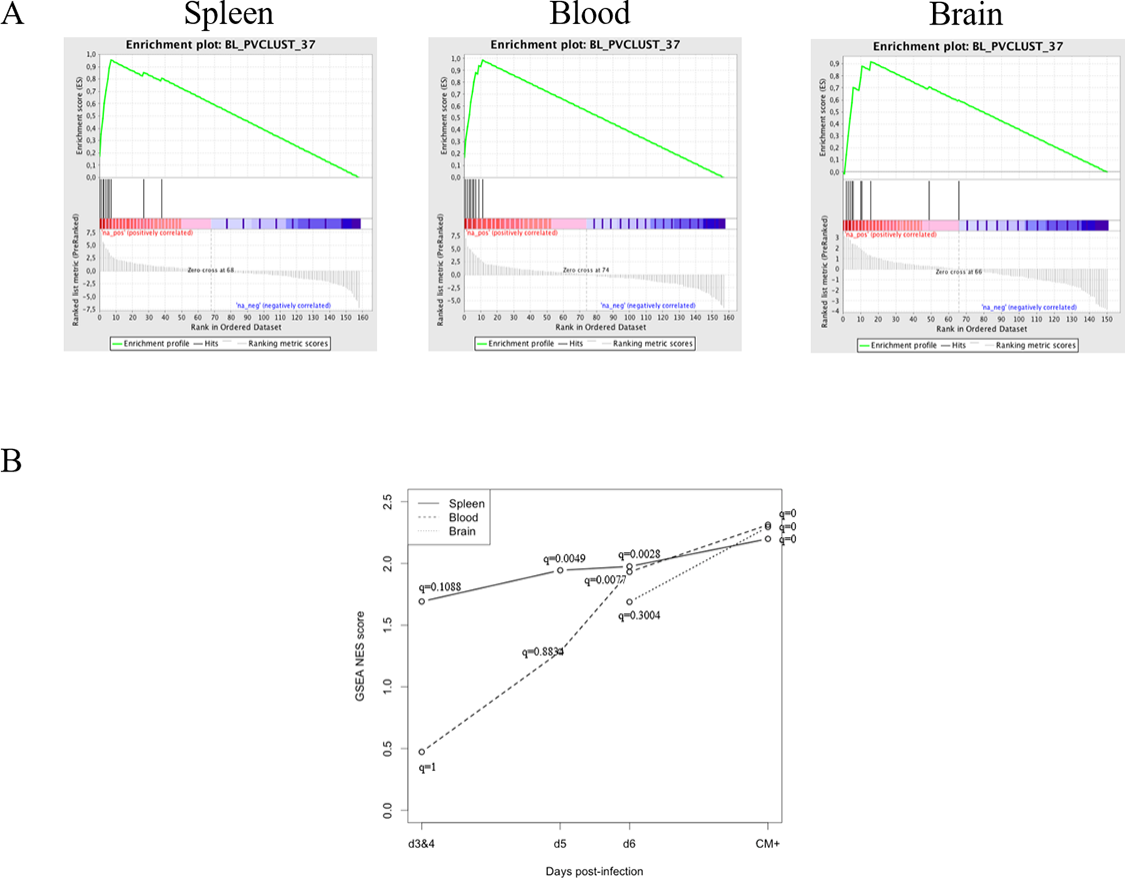
A unique TRBV signature discriminates CTR from CM^+^ spleen, blood and brain repertoires. (A) Panels of GSEA report for the peak set BL_PVCLUST_37 showing significant enrichment for CM^+^ condition compared to uninfected group in spleen (left) and blood (middle) and in the brain (right). The enrichment figures show ranked peaks according to the pooled t-statistic across imputed dataset (bottom). Peak positions are indicated on the ranked list (middle). The enrichment score (ES) is the maximum of the running sum (top). (B) Normalized GSEA enrichment scores post-infection growth curves in three compartments for the peak set BL_PVCLUST_37. In comparison to the CTR groups, the set is significantly enriched in day 5 post-infection in spleen (solid line), in day 6 in blood (dashed line) and in ECM state in brain (dotted line).

Furthermore, we tested the impact of ECM onset on TRBV peak composition. In other words, we looked for CM^+^ signatures as a combination of individual peaks taken separately from several TRBV that could predict the CM^+^ onset. The peak database was tested in GSEA software with pre-ranked option to determine whether our *a priori* defined peak sets are significantly enriched in the CM^+^ group. Peak sets are generated separately for each organ by pvclust and assembled within a peak set database. We retained clusters having at least 95% of confidence. In the presence of missing data, peak values are imputed using the multiple imputation process (see Material and Methods). Peaks were then ranked according to the pooled t-statistic value comparing uninfected samples to CM^+^ samples. GSEA reports (**Fig 5A**) identified a peak set that was significantly enriched in the CM^+^ group in the spleen (NES = 2.1744, q-value = 0), in the blood (NES = 2.3300, q-value = 0) and in the brain (NES = 2.2873, q-value = 0). This peak set contains peaks from TRBV1, TRBV3, TRBV4, TRBV5, TRBV12-2, TRBV13-2, TRBV19, TRBV26 and TRBV29 (**S2 Fig**) suggesting a rather restricted pathogenic T cell response. In order to assess the predictive power of this signature, we applied the process described in the methodology section separately for spleen and blood. For validation, we used previous data published in [22] as a test set to predict (“AC set”). As a training set, we used our current data from spleen and blood (“EMF set”). Due to a high number of missing data on the “EMF set”, we applied the multiple imputation process for peak data. Random forest models were trained on 20 CTR and 23 CM^+^ in spleen and on 20 CTR and 21 CM^+^ in blood and tested on 6 CTR and 9 CM^+^ in spleen and on 7 CTR and 13 CM^+^ in blood. Accuracy was assessed on train and test sets for each step. Random forest models showed very good accuracy > 90% of good prediction (**Table 2**). Altogether, the identified peak set allows to discriminate CM^+^ from CTR mice regardless of the organs, as validated on two independent datasets. We then asked whether the combinations of most discriminant peaks were different between organs, Random forest models providing score indexes to rank variables according to their importance. In the spleen, the most discriminant peaks are TRBV26_008, TRBV1_009, TRBV3_010 and TRBV5_010 whereas in the blood, the most discriminant peaks are TRBV1_009, TRBV19_006, TRBV26_008, TRBV5_010.

**Table 2:** Accuracy of random forest models.

Train on EMF, test on AC				Train on AC, test on EMF	
Prediction accuracy	Intermediate model Imputed train data	Final model Imputed train data	Final model test data	Final model test data	Final model train data
**Spleen**	97,00%	100,00%	100,00%	93,30%	92,70%
**Blood**	94,60%	99,56%	90,00%	95,00%	90,24%

To determine whether this signature can predict ECM outcome, we performed GSEA between each “kinetic” group and the CTR group in three compartments separately (due to small sample size, day 3 and 4 were pooled). During the course of infection, the peak set is significantly enriched at day 5 p-i in the spleen (NES = 1.9833; q-value = 0.0049), at day 6 p-i in the blood (NES = 1.9387; q-value = 0.0023) and in CM^+^ mice for the brain. Until day 6 p-i, NES is higher in spleen than in blood (**Fig 5B**), and then, it seems to converge to an equivalent value for the three compartments. These results are similar to those obtained from the analyses of global perturbation scores above. These findings reveal that the compartmentalized TCRβ repertoire changes can be detected at the level of TCR peaks. Moreover, the progression of NES in the spleen and in the blood underlines the diagnostic value of this TCRβ signature.

## Discussion

Cerebral malaria is a complex situation in which T cells are “necessary” to the development of the disease. Indeed, in the absence of T cells, ECM-susceptible mice are protected [8,42]. It is well described that CD8 T cells display a pathogenic role [12,13], mainly through their cytotoxic activity as shown by the protection associated with the perforin deficiency [43]. However, although up to seven target epitopes have been identified [15–17], none of them could definitely explain ECM development in mice [17]. A possible explanation is that as shown by Poh et al., part of these PbA epitopes are also shared by non-ECM inducing *Plasmodium* strains. An alternative explanation is that ECM occurs under the synergic/progressive presentation of several epitopes, including the CD8^+^ epitopes identified by the different studies but also still unknown CD4^+^ epitopes, given the critical role of CD4^+^ T cells in ECM development [8–10].

Here, we report a comprehensive analysis of the TCRβ repertoire during the course of PbA infection, including the analysis of brain T cell repertoires. Our results confirmed our previous observations of a massive perturbation of the repertoire associated with ECM onset, particularly in the blood. Furthermore, we showed that the modifications of the repertoire appear progressively in the spleen and in the blood, with TRBVs being differentially affected over time till all get perturbed. Strikingly, the comparison of brain repertoires between healthy and CM^+^ mice revealed a perturbation limited to three TRBVs out of 23. These three TRBVs are perturbed in the spleen and the blood, following a similar kinetic. These results indicate a targeted alteration of the brain TCRβ diversity, compared to the blood and the spleen. With regards to the important increase of cell numbers in the brain of CM^+^ animals, this suggests a selective recruitment of particular T cell clones in the brain directed against some particular parasitic or self-antigens.

As this is the first study showing the global repertoire of the brain T cells in naïve mice, we wanted to ensure that the CTR brain repertoire observed are not the result of blood contamination. In the case of CM^+^ brain samples, this can be excluded since most TRBV are perturbed in the blood when they are not in the brain. Surprisingly, no difference is observed between CTR blood and CTR brain repertoires. In a preliminary analysis, we observed that the blood repertoire is heterogeneous in CTR mice (data not shown). Since our perturbation index is a distance between each sample and an average reference repertoire, the DBV-BC values can differ depending on the reference repertoire used. Thus, we calculated the perturbation using CTR Blood as reference and observed that the brain T cell repertoire is indeed different compared to blood repertoire in naïve mice. This reflects the physiological compartmentalization of T cell diversity in naïve animals and highlights the importance of the microenvironment in shaping the TCR repertoire.

Finally, we could identify a TCRβ CDR3 length signature that discriminates CTR from CM^+^ repertoires in spleen, blood and brain. Using multivariate statistical modelling, we showed that this signature allows to discriminate the CTR and CM^+^ repertoires from spleen and blood obtained in a previous study, supporting the robustness and reliability of the approach. Thus, two phenomena seem to be involved in the disease. On the one hand, an alteration of the whole repertoire in spleen and blood could be associated with parasite mitogens or superantigens. Indeed, a superantigenic activity has been shown in PbA-infected C57BL/6 directing Vβ8.1 (TRBV13-3) expressing cells [20,21]. Additionally, a recent study on malaria-susceptible West-African children showed that the memory B cell repertoire is diverse, suggesting a response to several *Plasmodium* epitopes [44]. On the other hand, the identification of a unique and organ-independent TCRβ signature associated with CM^+^ onset suggests a response to specific antigenic peptides, possibly targeting antigens expressed in the cerebral compartment. The current hypothesis suggests that pathogenic T cells should be activated upon presentation of parasitic peptides [5]. Under these conditions, T cells could be selectively activated and attracted to the brain where they would play their pathogenic role, or have an autoimmune activity against brain molecules, through molecular mimicry, leading to brain damage. The recent identification of parasite-derived CD8 T cell epitopes supports this hypothesis [15–17]. Moreover, in those studies, CD8 T cells from CM^+^ mouse splenocytes also respond to the identified epitopes, again in line with our observations. Although the mouse strains used in our and the discussed studies are different, it can be noted that the TRBV signature we identified comprises the same TRBV genes as those found in response to the epitopes tested by Poh et al. and Howland et al. [15,17]. Moreover, we have shown that human CM is associated with an autoimmune blood B-cell repertoire, directed against a human brain protein [45], suggesting that *Plasmodium* can indirectly induce an autoimmunity-related process. These hypotheses are not exclusive and the activation site of pathogenic T cells remains to be elucidated. Our data suggest that the observed compartmentalization of the T cell repertoire reflects the selective migration of activated T cells from the spleen to the brain.

The complexity of the observed modifications is consistent with *Plasmodium* infection complexity. So far, therapies are oriented toward the development of vaccines targeting specific dominant antigens expressed by *Plasmodium*, and unfortunately all failed in inducing an efficient response against the parasite, as it has been confirmed in ECM by Poh et al. [17]. Finally, we identify a CM^+^ signature significantly enriched in the spleen and the blood during the course of the infection, strongly supporting the interest of diagnosis value of TCR repertoire studies. This study supports the effort made by several laboratory to analyse in parallel the modifications of the TCR repertoire diversity and the parasite antigenic variability to develop accurate and efficient therapies.

## Supporting Information

S1 Fig Random Forest prediction modelling approach. (TIF)

S2 Fig TCRβ signature peaks. (TIF)
