# Supplementary figures and images for: A TCRβ Repertoire Signature Can Predict Experimental Cerebral Malaria
Source: PLoS One. 2016 Feb 4;11(2):e0147871. doi: 10.1371/journal.pone.0147871 (PMC4742225; doi:10.1371/journal.pone.0147871)

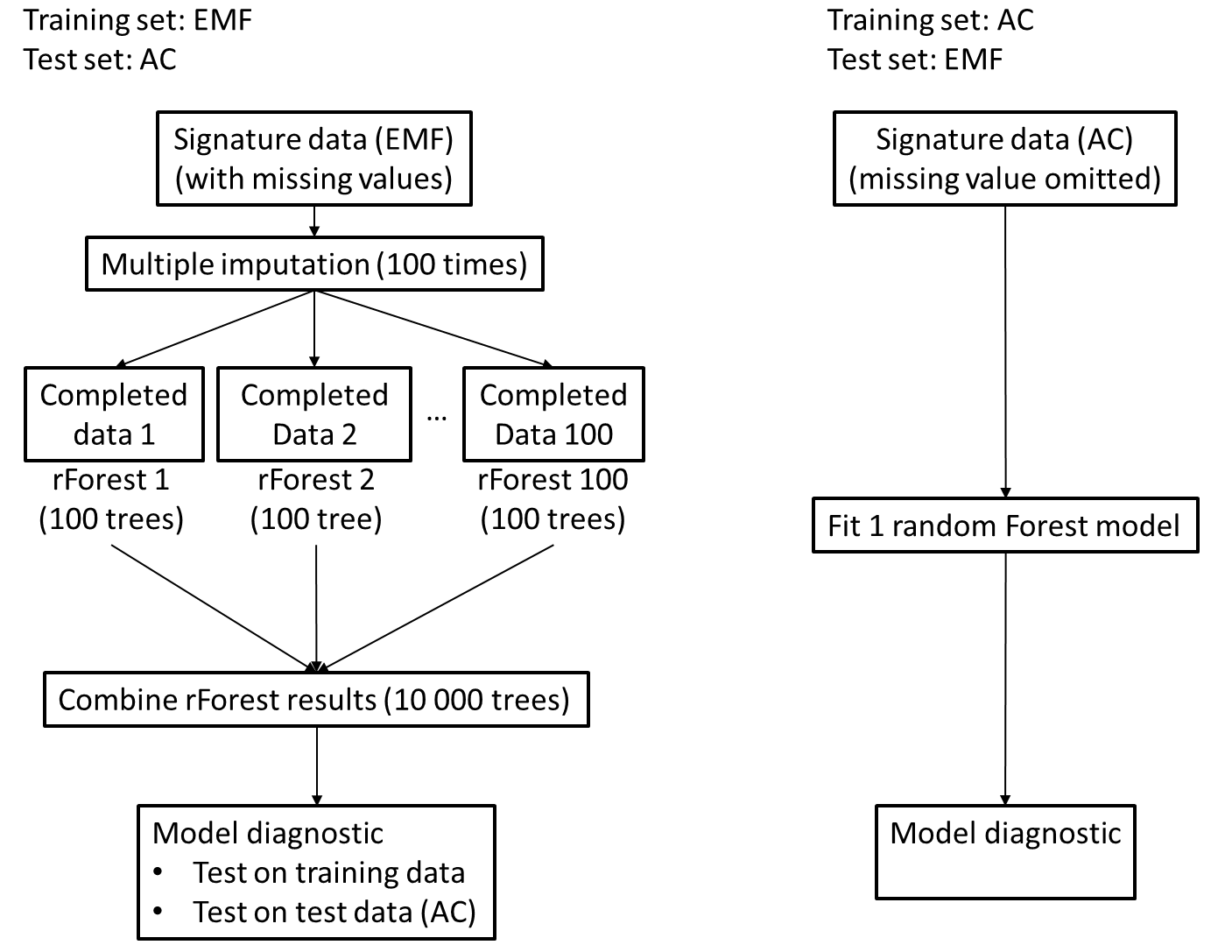

Supplement: S1 Fig — (TIF) [file pone.0147871.s001.tif]

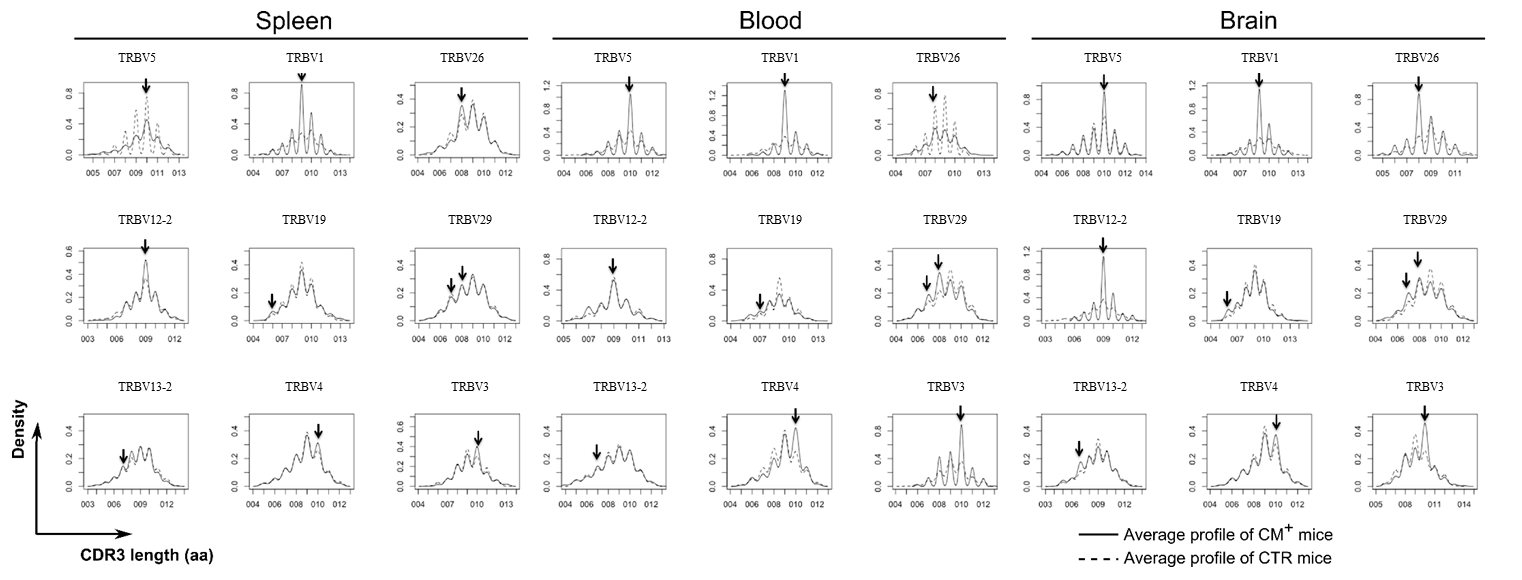

Supplement: S2 Fig — (TIF) [file pone.0147871.s002.tif]
